# The Ever-Evolving Concept of the Cancer Stem Cell in Pancreatic Cancer

**DOI:** 10.3390/cancers10020033

**Published:** 2018-01-26

**Authors:** Sandra Valle, Laura Martin-Hijano, Sonia Alcalá, Marta Alonso-Nocelo, Bruno Sainz

**Affiliations:** 1 Department of Biochemistry, Cancer Stem Cell and Tumor Microenvironment Group, Universidad Autónoma de Madrid (UAM), Madrid 28029, Spain; san.valle@predoc.uam.es (S.V.); laura.martinhijano@predoc.uam.es (L.M.-H.); sonia.alcala@uam.es (S.A.); mnocelo@iib.uam.es (M.A.-N.); 2 Department of Cancer Biology, Instituto de Investigaciones Biomédicas “Alberto Sols” (IIBM), CSIC-UAM, Madrid 28029, Spain; 3 Chronic Diseases and Cancer Area—Instituto Ramón y Cajal de Investigación Sanitaria (IRYCIS), Madrid 28034, Spain

**Keywords:** pancreatic cancer, cancer stem cells, plasticity, EMT, chemoresistance, autophagy, metastasis, hybrid cancer cell, metabolism

## Abstract

Pancreatic ductal adenocarcinoma (PDAC), the most common type of pancreatic cancer, is the 4th most frequent cause of cancer-related death worldwide, primarily due to the inherent chemoresistant nature and metastatic capacity of this tumor. The latter is believed to be mainly due to the existence of a subpopulation of highly plastic “stem”-like cells within the tumor, known as cancer stem cells (CSCs), which have been shown to have unique metabolic, autophagic, invasive, and chemoresistance properties that allow them to continuously self-renew and escape chemo-therapeutic elimination. As such, current treatments for the majority of PDAC patients are not effective and do not significantly impact overall patient survival (<7 months) as they do not affect the pancreatic CSC (PaCSC) population. In this context, it is important to highlight the need to better understand the characteristics of the PaCSC population in order to develop new therapies to target these cells. In this review, we will provide the latest updates and knowledge on the inherent characteristics of PaCSCs, particularly their unique biological properties including chemoresistance, epithelial to mesenchymal transition, plasticity, metabolism and autophagy.

## 1. Introduction

### 1.1. Pancreatic Cancer

**Figure 1:**
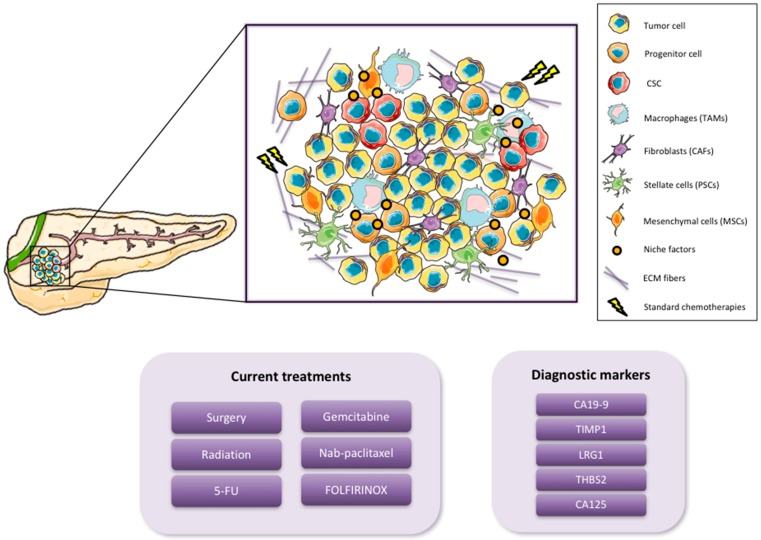
Pancreatic Ductal Adenocarcinoma (PDAC) tumor complexity, diagnosis and treatment. PDAC tumors include several cell types that interact with one another to maintain the tumor and facilitate PDAC progression. The tumor microenvironment plays a critical role in supporting tumor maintenance and expansion. The major cell types that belong to the PDAC tumor microenvironment are Tumor-associated macrophages (TAMs), Cancer-associated fibroblasts (CAFs), Pancreatic stellate cells (PSCs) and Mesenchymal stem cells (MSCs). Standard of care treatments only affect the tumor bulk, and diagnostic markers still lack sensitivity to adequately diagnose PDAC at early stages.

Pancreatic ductal adenocarcinoma (PDAC), the most common type of pancreatic cancer, is currently the fourth leading cause of cancer-related death in developed countries. By 2030, however, it is expected to become the second leading cause of cancer-related death, after non-small cell lung carcinoma, although its incidence will not increase significantly to surpass the five most common cancers, lung, breast, prostate, colorectal and bladder cancer [1]. These alarming statistics are due to the fact that there are no specific symptoms that can act as predictive indicators of this disease at an early stage. Likewise, the lack of early biomarkers and sensitive diagnostics only compounds the problem of early detection. As a consequence, the majority of pancreatic cancer patients are diagnosed when the disease is at an advanced stage and has already metastasized to distant secondary organs [2]. CA19-9 is the most common serum biomarker of disease progression used in the clinic; however, it is not recommended for general screening as this marker varies with the disease stage, and it is prone to false positives [3]. For this reason, new serological markers complementing CA19-9 are urgently needed in order to detect PDAC at early stages when potentially curable surgical resection is still possible. While molecular markers could provide more specific information for patients, their development and implementation still remain a challenge and in general has been a slow process [4]. Recently, candidate biomarkers have been identified which could complement CA19-9 for detection of PDAC at earlier stages. Addition of tissue inhibitor matrix metalloproteinase 1 (TIMP1), leucine-rich-α-2-glycoprotein1 (LRG1) or thrombospondin-2 (THBS2) to CA19-9 screening has been shown to significantly improve early PDAC tumor detection [5,6]. In addition, a new marker, CA125, has also been found and shows an encouraging sensitivity for the preclinical detection of this disease [7] (Figure 1).

Pancreatic cancer usually presents in the head of the pancreas and extends through the surrounding tissue and metastasizes to the liver, spleen and peritoneum [8]. In general, PDAC has a higher incidence in men, increases with age and predominates in certain racial groups [9]. Increased risk is likely due to a combination of both environmental and genetic factors. Smoking, for example, has been identified as a clear risk factor, as it has been shown that smokers are up to three times more likely to develop pancreatic cancer. In addition, diabetes, obesity and chronic pancreatitis also confer an increased risk for the development of PDAC [10,11]. Current statistics show a progressive increase of new PDAC cases every year. More than 53,000 new cases occur annually and pancreatic cancer continues to cause 41,000 deaths each year in the United States [12]. Furthermore, less than 7% of patients achieve long-term (>5 year) survival [13]. Surgical resection seems to be the only potentially curative therapy for patients with PDAC; however, only 15–20% of patients are eligible, and within this subset of patients, only 15–25% have an overall survival rate of greater than 5 years [14,15]. Thus, while successful early detection is necessary and has been shown to be a significant contributor in altering both the incidence and death rates in other solid tumors, the fact remains that the vast majority of PDAC patients are diagnosed with advanced-stage non-resectable disease. As such, the current reference treatments, which include 5-fluorouracil (5-FU), gemcitabine, albumin-bound paclitaxel (nab-paclitaxel) plus gemcitabine, or FOLFIRINOX (folinic acid, 5-FU, irinotecan, and oxaliplatin), only prolong the survival of patients by 6–12 months, while having essentially no real effect on tumor eradication [16] (Figure 1). Nevertheless, substantial advances achieved in the last two decades with respect to better understanding the biology of pancreatic cancer have shed light on the cellular complexity, composition and heterogeneity of this disease. Along these lines, it is now universally accepted that PDAC metastasis, chemoresistance, and disease relapse is driven by a subpopulation of highly plastic “stem-like” cells within the tumor known as cancer stem cells (CSCs), and from a clinical perspective, understanding how these cells function and developing inhibitors to eliminate the CSC population should lead to tumor eradication [17,18]. Importantly, CSCs co-exist with other cellular components that constitute the tumor microenvironment (TME) [19], thus understanding the TME and the dynamic cross talk between CSCs and the TME is also equally important. One of the main characteristics of PDAC is its desmoplastic microenvironment, which is composed of a very dense and highly organized collagen fibers, such as collagen type V [20,21]. In addition, the PDAC TME includes cancer-associated fibroblasts (CAFs), pancreatic stellate cells (PSCs), mesenchymal stem cells (MSCs) and immune cells such as macrophages [19] (Figure 1). All of these cellular and non-cellular components fulfill different roles during PDAC tumorigenesis, evolution and progression such as development support, chemoresistance and CSC activation [22,23].

### 1.2. Cancer Stem Cells

Over the years, several models have been proposed to explain the heterogeneity, chemoresistance and aggressiveness of pancreatic tumors. The two most widely studied models have been the clonal evolution “stochastic” and the CSC “hierarchical” models. While each model is different, we now appreciate that both models are not mutually exclusive, and both can be interrelated [24,25]. The main difference between the two models is that the CSC “hierarchical” model proposes that a particular subset of tumor cells, with “stem-like” properties, are the sole drivers of tumor formation [26], metastatic dissemination, chemoresistance, and disease recurrence/relapse [27]. These cells have unlimited self-renewal capacity and can differentiate into all of the cancer cell populations present within the tumor, thus CSCs represent the cell population uniquely responsible for tumor heterogeneity. Tumor heterogeneity is a very complex process and one that is still not entirely understood. Based on the CSC model and assuming that the CSC is an “entity”, it is believed that a tumor contains several CSC clones that are genetically and/or epigenetically different. Each different CSC subclone could then give rise to intermediate progenies or transitory/hybrid cells [25] that would drive the expansion of the bulk cancer cells present within the tumor; however, these progenies would lack the stem-like properties inherent to the CSC from which they were derived, such as unlimited self-renewal capacity. At any given time, and based on intratumoral or extrinsic pressures such as nutrient deprivation, hypoxia, or chemotherapy, specific CSC clones can dominate while others can essentially disappear. The existence of silent or undetectable CSC clones was demonstrated by Dieter et al. [28] in an elegant study aimed at tracing tumor-initiating cells (TICs) from human colon cancer tumors and metastases. Using a molecular tracking strategy in which sphere-derived colon cancer cells were infected with lentiviral vectors, the authors were able to amplify, by linear amplification-mediated PCR, clone-specific insertion site fusion sequences in order to trace and detect TICs. Following serial transplantation of cells from primary to tertiary mice, the authors discovered three types of TICs: extensively self-renewing long-term TICs (LT-TICs), tumor transient amplifying cells (T-TACs) and rare delayed contributing TICs (DC-TICs). While LT-TICs possessed long-term self-renewing capacity, and were responsible for metastasis formation, T-TACs had limited self-renewal capacity and DC-TICs represented a silent subpopulation of cells that were active during secondary and tertiary transplantation. Thus, while tumor formation and metastases are largely driven by a specific population of TICs, rare delayed TICs also exist and can dominate under specific conditions. On the other hand, if we assume that the CSC is a “state”, then daughter or progenitor cells that continue to accumulate genetic changes during tumor proliferation or that alter their epigenetic programming via interactions with the TME could acquire a mutation or epigenetic mark that would confer upon them CSC characteristics [29], resulting in the generation of a new CSC clone at any given time during the life of the tumor. The idea of the CSC as an “entity or a state” will be further discussed below. 

**Figure 2:**
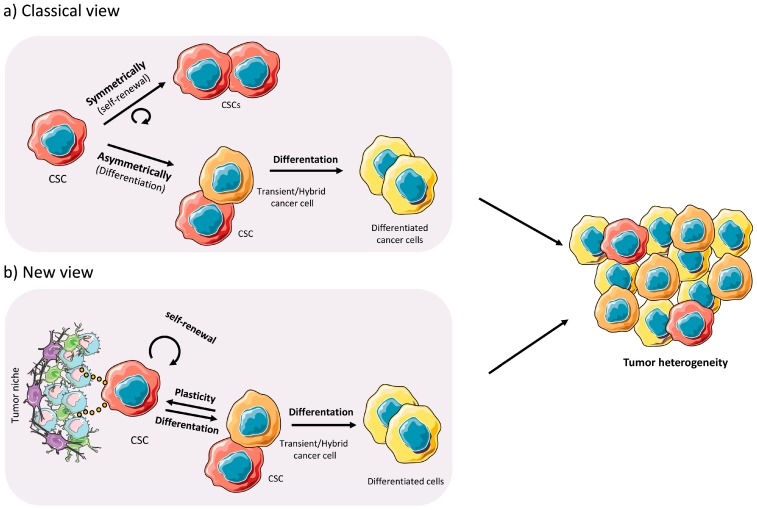
Models of the cancer stem cell concept to explain tumor heterogeneity. (**a**) Classical view shows the two pathways of Cancer Stem Cells (CSCs) differentiation: symmetrically (self-renewal) resulting in two CSCs and asymmetrically (differentiation) giving rise to a CSCs and a transient/hybrid cancer cell that gives rise to the more differentiated cells. These more differentiated cells divide rapidly and have less tumorigenic potential and plasticity compared to CSCs or transient/hybrid cancer cells. (**b**) Nowadays, this classical model has undergone some modifications. CSCs receive necessary signals from the tumor niche/tumor microenvironment (TME) modulating the path taken for their division. Due to the plasticity of the transient/hybrid daughter cells, they can also undergo reprogramming into a CSC via signals received from the tumor niche/TME. In addition, transient/hybrid cells can also give rise to differentiated bulk cancer cells.

For the CSC model to function, however, the CSC pool must never be extinguished and functional/biological properties must exist to prevent their loss. Indeed, CSCs have the ability to renew themselves indefinitely via asymmetric and symmetric division. Asymmetric (differentiation) division gives rise to a CSC and a differentiated tumor cell, while symmetric (self-renewal) division gives rise to two identical CSCs. In both cases, the self-renewal capacity remains intact, which ensures the pool of CSCs and supports the hierarchical model of tumor cell heterogeneity, with the CSC being the cell solely responsible for the formation of tumors, while non-CSC cells lack this capacity [30] (Figure 2a). In addition, CSCs possess other properties that ensure their survival, which include inherent resistance to chemotherapeutics, multipotency and the capacity to metastasize [31]. What continues to elude investigators is why this subpopulation of cells possess these unique properties, while non-CSCs do not. The existence of specialized cells with phenotypic plasticity is important during embryogenesis, and to some extent adult stem cells possess this behavior. In cancer, reactivation of specialized embryogenic pathways is observed, and has been shown to drive CSC genesis and tumor formation [32]. Therefore, it is believed that CSCs are epigenetically and metabolically distinct from their non-CSCs and these modifications are necessary to facilitate their phenotypic plasticity [33] (Figure 2b). These concepts will be discussed in further detail below. 

In summary, the CSC concept is well accepted and believed to be the best model to understand tumor heterogeneity and plasticity. While first proposed more than 150 years ago [34], the CSC hypothesis would not be officially accepted until 1994 when Dick and colleagues formally proved their existence in hematological malignancies thanks to the advent of fluorescence-activated cell sorting combined with in vivo models of tumor growth in immunodeficient mice [35]. Since then, CSCs have been identified in essentially all solid tumors, including pancreatic cancer [36,37], and their existence has been directly linked to tumor progression, poor prognosis, disease recurrence, and metastasis [38]. These cells are dispersed within the tumor mass and represent only a small percentage of the total number of cancer cells present within the tumor [19], making their identification, isolation and study challenging. Nonetheless, it is essential that we further develop methodologies that allow us to distinguish CSCs from other tumor cells in order to develop new therapies that can specifically target and eliminate this cellular subpopulation and thus prevent tumor recurrence and metastatic disease.

## 2. Pancreatic CSC (PaCSC) Characteristics

Since their discovery in 2007 [36,37], pancreatic CSCs (PaCSCs) have been identified, isolated and dissected using a variety of biomarkers. The most widely used strategy for the identification and study of CSCs is based on the use of antibodies directed against certain cell surface antigens such as CD44, CD90, CD24, CD133, LGR5, EpCam, and/or CXCR4 [36,37,39,40]. Other markers have also been used to identify and isolate PaCSCs including 26S proteasome activity [41], aldehyde dehydrogenase 1 (ALDH1) [40], autofluorescence [42] and hepatocyte growth factor receptor C-MET [43]. While the use of these markers has increased our underlying knowledge of the biology of PaCSCs and has facilitated the identification of common features across many types of cancer, such as unlimited self-renewal capacity, exclusive tumorigenicity or inherent chemoresistance, the molecular mechanisms driving the stem-like state of PaCSCs is still incomplete and requires further investigation. We know that at the molecular level, the PaCSC subpopulation differs from that of non-CSCs, and these differences are present at multiple levels and involve numerous biological aspects, such as genetics, epigenetics, protein modifications and signaling networks. Thus, any biological feature that makes CSCs stand out over the rest of the tumor cells, as well as from normal stem cells, could provide a potential target for either eliminating or disrupting the CSC subpopulations. Below we will review the main characteristics and biological features of PaCSCs, discuss new concepts in the CSC field, including plasticity, and highlight key studies that have attempted to dissect the drivers/mediators of stemness in PaCSCs, with the goal of widening our understanding of their unique biology in order to improve our capacity to target them and thus facilitate tumor eradication.

### 2.1. Chemoresistance

It is well known that PaCSC have a strong and inherent chemoresistant profile. In fact, chemoresistance, together with metastatic potential and relapse, are the main clinical hallmarks of PaCSCs. While intrinsic chemoresistance exists prior to treatment (e.g., ERBB2 overexpression [44,45] or constitutive nuclear factor kappaB activity (NF-κB) [46]) acquired chemoresistance is induced following treatment [47]. As reviewed in Abdullah and Chow [48], CSCs are able to evade the cytotoxic effects of chemotherapeutic agents via diverse inherent and acquired mechanisms, such as the over-expression of ATP-binding transporters (ABC transporters), pro-survival B-cell lymphoma-2 (BCL-2) protein family members, ALDH1 isoforms that confer cyclophosphamide resistance, the activation of CSCs-related signaling pathways including MYC, AKT1, WNT/β-catenin, Notch and Shh, and an enhanced DNA damage response. Some of these mechanisms will be further discussed below.

ABC transporters, such as multidrug resistant protein 1 (MDR1) or ABCG2, are known effectors of chemoresistance across multiple tumor types [49]. Over expression of these transporters in PaCSCs creates a highly efficient efflux pump system compared to non-CSCs, resulting in prompt elimination of chemotoxic agents. Surprisingly, ABCG2 localization and activity is not confined only to the plasma membrane. Miranda-Lorenzo et al. [42] discovered the presence of autofluorescent vesicles within the cytoplasm of CSCs of PDAC, colorectal cancer and hepatocellular carcinoma. The authors were able to establish a direct relationship between the presence of these vesicles in CSCs and the maintenance of their stem-like properties, including chemoresistance. These vesicles were shown to accumulate ABCG2-dependent substrates, such as the fluorescent vitamin riboflavin (vitamin B2), due to the overexpression of ABCG2 transporters on the membrane of these vesicles. Moreover, the authors also showed that these autofluorescent vesicles could accumulate ABCG2-depedent therapeutics, such as mitoxantrone, to avoid apoptotic cell death. This finding provides not only a potential target to modulate CSC chemoresistance, but also a useful biomarker for the detection and study of CSCs across different tumor entities. Another typical CSC marker that also functions as a mediator of drug resistance is ALDH1. ALDH1 is a member of a family of intracellular cytosolic enzymes involved in cellular detoxification and differentiation through the oxidation of aldehydes to carboxylic acids and the transformation of retinol into retinoic acid, respectively, and its activity has been linked to normal multipotent stem and progenitor cells (reviewed in [50]). While ALDH1 has been used as a CSC marker in several types of solid neoplasms, including breast, lung, prostate, brain, liver, gastric, colorectal cancer and PDAC [40,51,52,53,54,55,56,57], ALDH1 has been shown to mediate cyclophosphamide- as well as gemcitabine-resistance in PDAC [58]. For example, using gemcitabine resistance as a CSC hallmark, Duong et al. [58] showed that ALDH1 silencing reduced PDAC cell proliferation and increased their sensitivity to treatment with gemcitabine. ALDH1 and autophagy have also recently been associated. A study by Yang et al. [59] demonstrated that the expression of the autophagy factor LC3 in pancreatic cancer tissues positively correlated with the PaCSC markers ALDH1, CD44, and CD133. Likewise, they showed that high co-expression of LC3/ALDH1 was associated with survival, and inhibition of autophagy reduced the ALDH1 CSC population and their resistance to gemcitabine in vitro and in vivo. The role of autophagy in PaCSCs will be discussed in more detail below. Moreover, another mechanism by which CSCs resist standard treatments is via an augmented DNA damage response, particularly for radiotherapy or genotoxic drugs [48]. 

**Figure 3:**
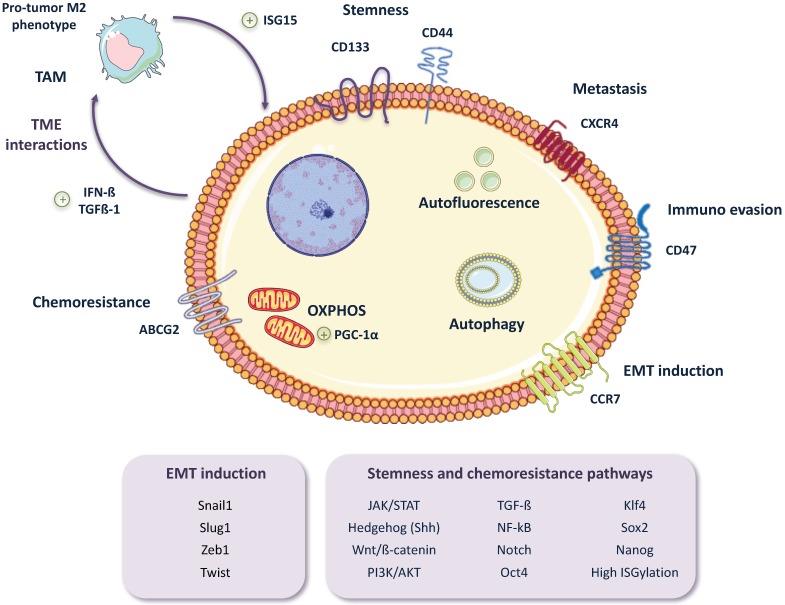
Pancreatic CSCs features. Pancreatic CSCs (PaCSCs) include various CSC subpopulations with distinct characteristics. This figure shows some of the PaCSCs features, which may not be shared between all subpopulations. PaCSCs can express several markers for stemness, metastatic potential, immuno evasion and chemoresistance, as well as receptors for epithelial to mesenchymal transition (EMT) induction. Oxidative phosphorylation (OXPHOS) is a key feature of PaCSCs, as is enhanced autophagy and the accumulation of autofluorescent vesicles. Numerous stemness and chemoresistance signaling pathways may also be activated, strengthening CSC survival and PDAC progression, as well as relapse after treatment. JAK/STAT (Janus tyrosine Kinase/Signal Transducer and Activator of Transcription); PI3K (phosphatidylinositol 3-kinase); PGC-1 α (Peroxisome proliferator-activated receptor γ co-activator 1 α).

While there have been little advancements overcoming PDAC chemoresistance in the clinic, new insights into the molecular processes underlying this phenomenon are beginning to emerge as a result of intensive research. What we are discovering is that chemoresistance is much more than the overexpression of drug efflux transporters or the modulation of the cell’s DNA damage response, but rather many signaling pathways related to the stem-like phenotypes of CSCs are also intimately linked to CSC chemoresistance. Such is the case for Hedgehog (Hh), NF-κB, Notch, AKT1, Tumor growth factor-beta (TGF-β) or Wnt/β-catenin signaling pathways, all of which are both signaling pathways upregulated in CSCs and pathways that can promote chemoresistance [60,61,62,63,64,65,66,67,68] (Figure 3). Along these lines, CSCs overexpress or silence numerous pathways and/or factors in order to enhance their inherent resistance to different chemotherapeutics. For example, a recent study using chemoresistant CSCs derived from Panc-1 and PSN-1 cell lines discovered that JNK, a key regulator of CSC maintenance that is up-regulated in PaCSCs [69], appears to mediate both gemcitabine and 5-FU resistance. The authors suggest that over-activation of JNK may be blocking the increase of intracellular ROS levels induced by gemcitabine or 5-FU, thus inhibiting apoptosis and CSC death [70]. Another factor that promotes both stem-like features and chemoresistance in PDAC, by activating TGF-β and Wnt/β-catenin signaling, is FAM83A, a member of the PLDc_SuperFamily. Chen et al. [71] showed in both in vitro and in vivo studies that gene amplification and over-expression of FAM83A in patients correlated with poor overall survival, and its inhibition in PDAC cell lines and mouse xenografts resulted in a reduction of stem-like features and enhanced sensitivity to gemcitabine. Moreover, Ina et al. [72], through a microarray analysis of 11 PDAC cell lines, observed that Interferon-Stimulated Gene 15 (ISG15) directly correlated with gemcitabine-resistance. While an innate immune response protein, ISG15 is an ubiquitin-like protein that modifies proteins via a process known as ISGylation. Thus, ISG15 may be promoting chemoresistance in PDAC via a downstream protein modification process similar to ubiquitination. 

DNA methylation, histone acetylation and miRNA (miR) expression can also regulate factors involved in CSC chemoresistance. PaCSCs and non-CSCs share the same genetic background; therefore, differences in the epigenetic regulation of gene expression are in great part responsible for their differential features, including chemoresistance. For example, Jiang et al. [73] showed that miR-1181, a negative regulator of the CSC-related genes *SOX2* and *STAT3*, is downregulated in human pancreatic cancer tissues and cell lines. While low levels of miR-1181 correlated with poor overall survival in patients and promoted the stem-like state of PaCSCs in vitro and in vivo, overexpression of miR-1181 inhibited CSC-like phenotypes including chemoresistance [73]. Along these lines, a very illustrative study by Cioffi et al. [74] showed a specific downregulation of the miR-17-92 cluster in gemcitabine-resistant PaCSCs versus non-CSCs, revealing a determining role for this miRNA cluster in chemoresistance, as well as in PaCSC self-renewal and tumorigenicity. The miR-17-92 cluster is an inhibitor of the NODAL/ACTIVIN/TGF-β1 axis, which is a major promoter of stemness and a critical player in the metastatic capacity of PaCSCs [32]. The authors show that some of the members of this signaling axis are directly inhibited by this cluster, such as p21 and p57, both of which are known to be regulators of quiescence [75,76], and TBX3, which has been shown to regulate self-renewal in embryonic stem cells and breast CSCs [77]. The specific downregulation of the cluster in PaCSCs is a clear example of the epigenetic regulation of chemoresistance in PaCSCs. The same group published a year later that the regulation of this cluster in PaCSCs versus non-CSCs is due to DNA methylation levels. The study by Zagorac et al. [78] revealed that PaCSCs possess higher DNA methylation levels compared to non-CSCs, via up-regulation of DNMT1. In accordance with their previous work, the miR-17-92 cluster was among the genes silenced, and global demethylation with compounds such as Zebularine could increase the expression of the miR-17-92 cluster family members, affecting “stem-like” traits including differentiation and drug resistance. In addition, selective demethylation of certain genes involved in stemness may also occur in PaCSC, as in the case for *SOX9*. The hypomethylation of the *SOX9* promoter allows the expression of this stemness gene, involved in invasive phenotypes and which has been shown to be directly activated by NF-κB, which is over-activated in PDAC cells [79]. In light of these findings it seems clear that there are different layers regulating PaCSC chemoresistance, implying the need for a combination of different approaches to successfully reach the CSC population and chemosensitize these cells. It is important to note, however, that although many of these mechanisms of chemoresistance can be shared among several different CSC types, they are not universal for all CSCs and many of them seem to be cancer-specific, what can be due at least in part to the tumor characteristics, such as the TME, vascularization, hormonal influences or stromal composition. In this sense, CSCs have been reported to remodel the tumor microenvironment [80] including the stroma, either indirectly through promoting the generation of CAFs [81], or directly as has been described in liver [82] and breast cancer [83]. The influence of PaCSCs on stromal composition and remodeling has not been fully elucidated, although a study by Shimizu et al. [84] describe ECM remodeling by PaCSCs in vitro. Therefore, further research is necessary to better understand the role of PaCSC in this phenomenon. For this reason, it is essential to dissect mechanisms and cellular components of chemoresistance and unravel each specific chemoresistance feature in the most appropriate cancer system and model.

### 2.2. EMT, Invasiveness and Metastasis

Epithelial-to-Mesenchymal Transition (EMT) is a process whereby epithelial cells undergo numerous biological changes that confer a mesenchymal phenotype upon them. It includes the gain of migratory and invasive capacities, high resistance to apoptosis and increased secretion of extracellular matrix (ECM) components. It is mainly orchestrated by the expression of a battery of transcription factors, including Snail, Slug, zinc finger E-box binding homeobox 1 (Zeb1), Twist, Goosecoid, and FOXC2 (reviewed in [85]). This fine-tuned regulation of gene expression is also strongly supported by the miRNA machinery [85]. EMT is essential for physiological processes like embryogenesis, wound healing and tissue regeneration; however, it is also involved in pathogenic processes such as fibrosis or cancer development. While this review focusses on EMT, it is important to note that other processes similar to EMT exist and also likely play an important role in PDAC. For example, endothelial-to-mesenchymal transition (EndMT) is a subtype of EMT, and it is also associated with fibrosis in different pathologies, including cancer, and EndMT has also been linked to CSC stemness [86]. As described by Matkar et al. [87], EndMT plays an essential role in tumor fibrosis and PDAC aggressiveness. The authors showed that blockade of Neuropilin-1 (Nrp-1) in vivo reduced TFGβ-mediated EndMT, fibrosis and tumor size. Moreover, Nrp-1 is an important factor for tumor progression, which is overexpressed in PDAC [88]. In addition, EndMT is responsible for the formation of up to 40% of CAFs [89], and as mentioned above, CAFs are key components of the TME and are involved in stroma remodeling and the secretion of oncogenic factors [90]. We refer the reader to the following review on EndMT for additional information [91].

Regarding EMT, intensive research has focused on elucidating the drivers and molecular mechanisms of EMT in cancer, especially in order to understand its role in and relationship with metastasis. In the case of PDAC, the idea that EMT is a driver of PaCSC metastasis has been well accepted for decades. However, in 2015, Zeng et al. [92] showed that *SNAI1* and *TWIST1* deletion in the KPC (LSL-Kras(G12D/+); LSL-Trp53(R172H/+); Pdx-1-Cre) mouse model of PDAC resulted in a significant decrease in EMT-associated gene expression, a reduction in the expression of the EMT marker αSMA in epithelial cells, and an increase in the expression of the epithelial marker E-cadherin; however, no effect on either metastasis nor invasiveness, two phenotypes strongly associated with EMT, were observed. Interestingly, the authors did discover that Snail1 and Twist1 are critical for PDAC chemoresistance and cell proliferation. In light of these results, the authors claimed that EMT was dispensable for PDAC metastasis, a revolutionary concept at the time. In 2017, however, Krebs et al. [93] showed that genetic depletion of *ZEB1* in the same KPC model used by Zheng and colleagues led to a decreased expression of the EMT inducer factors Zeb2, Slug and Snail, but not Twist1. Moreover, *ZEB1* depletion resulted in a more differentiated state of tumor cells and an increased expression of Gata6, a marker of differentiation in PDAC [94]. More importantly, *ZEB1* deletion had a significant and negative effect on tumor progression, invasiveness, and metastasis, reaffirming EMT’s role in PDAC metastasis. The authors therefore described “tissue-specific roles for EMT transcription factors, that may play non-redundant roles, and must be taken in consideration when dissecting EMT functioning”.

As mentioned above, the miRNA machinery also plays a critical role in EMT regulation. A recent study from Tsukasa et al. [95] showed an interesting connection between CD133, a known PaCSC marker, and the activation of EMT through the miR-30 family in the pancreatic cancer cell line Capan-1M9. The functional link between CD133 expression and the activation of mesenchymal markers, as well as the switch of E-cadherin to N-cadherin, had been previously shown by this group [96]. Based on those findings, they performed a genetic knockdown of CD133 and observed a reduction in the expression of the miR-30 family. In addition, the over expression of miR-30 resulted in an increased expression of mesenchymal markers such as fibronectin, vimentin and N-cadherin, and promoted migratory and invasive phenotypes, as well as gemcitabine resistance [95]. Therefore, CD133 seems to be not only an extremely useful marker for CSCs, but also a contributor to the activation of the EMT program and thus a driver of the stem cell “like” state of CSCs (Figure 3). 

EMT has been closely associated with stem-like properties, and in particular to de novo CSC-genesis, as it has been shown to induce stem-like properties in different cancers [97,98,99]. This induction has been intensively studied in breast cancer, and numerous authors have reported the activation of the CSC state via EMT. For example, Mani et al. [100] showed that EMT induction in immortalized human mammary epithelial cells activated the expression of stem cell markers and increased their capacity to form mammospheres and tumors in vivo. Moreover, mammospheres cultured from the same mammary epithelial cells expressed similar markers to the cells that had undergone EMT, suggesting a direct association between EMT and the development of CSCs traits. Similar results were obtained in a study of Gupta et al. [52], in which *CDH1* silencing or *TWIST1* expression in HMLER breast cancer cells led to EMT activation and a subsequent increase in the CSC fraction and stem-like properties, such as higher mammosphere formation ability and increased tumorigenesis in vivo. In addition, Xie et al. [101] revealed that IL-6-induced EMT promoted the generation of CSCs in breast cancer cells. In contrast, the induction of the CSC state by EMT remains unclear and less defined in PDAC, although some recent studies would suggest a similar link between EMT and stemness, chemoresistance and metastatic capacity [102]. For example, a recent study by Zhang et al. [103] illustrated the importance of the CCR7/CCL21 axis in promoting an EMT phenotype in PaCSC derived from Panc-1, AsPC-1, and MIA PaCa-2 cell lines, which have a very high invasive potential. Higher levels of CCR7 were found in CD133^+^ PaCSC, PDAC tumor tissues and metastatic lymph nodes, compared to CD133^−^ cells, adjacent normal tissues from patients with PDAC and normal lymph nodes, respectively. Moreover, activation of signaling from CCR7/CCL21 promoted PaCSC survival via ERK/NF-κB and metastatic potential via EMT induction. Important stemness signaling pathways have been related to metastasis in PDAC, such as Hh signaling. Hh pathway-specific roles in PaCSCs were defined in a study based on the knock-down of Smoothen in Panc-1-derived CSCs and in a nude mouse lung metastasis model. Disruption of Hh signaling in PaCSCs resulted in a reduction of self-renewal, EMT, tumorigenesis, invasiveness, chemoresistance and pulmonary metastasis [104]. More recently, Shi et al. [105] reported the important role of Cyr61, a modulator of cell adhesion, DNA synthesis, cell survival and angiogenesis, in PDAC metastatic spread. Suppression of Cyr61 resulted in a reduction of the CSC population in vitro, as well as the reduction of tumor growth, tumorigenicity and liver and lung metastases in vivo. Moreover, higher levels of Cyr61 were found in both primary PDAC and metastasis samples from patients with distant metastasis at the time of diagnosis compared to those without. Although the mechanism of action of this factor is still not fully understood, it seems to activate the PI3K signaling pathway, thus promoting cell proliferation, chemoresistance and, importantly, invasiveness.

Whereas EMT has classically been considered the cornerstone of metastasis initiation, Mesenchymal to Epithelial Transition (MET) has been described as crucial for seeding stages. Recent studies demonstrate the existence of a hybrid epithelial/mesenchymal phenotype in cells transitioning between EMT and MET, that involves mixed epithelial and mesenchymal features (e.g., adhesiveness and motility). This phenotype seems to be linked to drug resistance and tumor-initiating potential; moreover, it allows tumor cells to collectively migrate in clusters to form metastases in a more effective way than pure EMT single cells [106]. Thus, our concept of EMT and MET as binary processes has changed, and EMT appears now as a continuum or spectrum, that comprises different stages between the pure epithelial and mesenchymal states [107]. In fact, many groups now refer to cells as epithelial (E), intermediate epithelial (iE), hybrid E/M, intermediate mesenchymal (iM) and mesenchymal (M). This new paradigm (discussed in more detail below) has enabled us to better understand the complex dynamics of the metastatic processes, and the recognition of the described hybrid E/M state and its properties will surely lead to a better conceptual dissection of the stages and development of metastasis. It seems clear that the tumor circulating cells (CTCs), present in the bloodstream and that mediate metastasis, should have EMT and tumor-initiating properties (i.e., CSCs traits that would allow them to successfully metastasize and rebuild the original tumor at secondary distant sites). Thus, it is not surprising to find a highly metastatic CSC sub-compartment within the CTC population [108,109,110] with E/M hybrid properties that set them apart from other CTCs [111]. Based on these studies, it seems clear that a CSC population with metastatic potential, acquired mainly via EMT and/or TME cues, is the driver of metastasis in several cancers, and PDAC is likely not an exception. Hermann et al. [37] showed that a subpopulation of PaCSCs expressing the markers CD133 and CXCR4 were responsible for metastasis formation in vivo, and discussed how hypoxic conditions in the TME can induce a hypoxia-inducible factor (HIF)-dependent activation of CXCR4. An interesting study by Poruk and colleagues [110] using PDAC CTCs from 60 patients revealed the presence of a CSC compartment in the majority of them. Patients with CTCs expressing a cytokeratin epithelial marker and the CSC markers CD133 and/or CD44 had a higher risk of tumor recurrence and lower overall survival, compared to those with cytokeratin^+^/CD133^−^/CD44^−^ CTCs. Considering that there was no way to track the CSC-like CTCs, it was impossible to determine whether the CSC subpopulation was responsible for distant metastasis formation. The mapping of subclones in the primary tumor, CTCs and metastasis would provide the link needed to support such claims. In a PDAC mouse model, Maddipati and Stanger [112] conducted an illustrative and elegant study using multi-color lineage tracing technology to unravel the cellular dynamics of metastasis in vivo. This system allowed them to examine the heterogeneity of primary tumors from the first initial stages of tumor development, to large metastasis development in the peritoneum and diaphragm and finally to micro-metastases formation in the liver and lung. All metastatic lesions appeared to be polyclonal, and were comprised of a maximum of 2 populations. Ascites fluid or blood analyses revealed the presence of bi-chromatic clusters of tumor cells, supporting the idea that the seeding cells were polyclonal from the beginning, and not the two-step mechanism by which the seeding of the first clone would be followed by the recruitment of the second one. This idea was reinforced by the fact that clusters of cells were more efficient at forming metastases than sorted single cells of either subclone. Moreover, they ruled out the possibility of clusters forming in the bloodstream by sequentially injecting the two sorted populations, and comparing the composition of metastatic lesions with those formed after injecting clusters; however, although the initial steps seem to be equal for all types of metastases found, lung and liver metastatic lesions would rapidly lose their polyclonality and become dominated by a single clonal population. The conclusion of this study supports the concept of metastasis as a result of tumor subclone interactions that lead to the dissemination of tumor cellular aggregates, rather than the classical idea of a single cell undergoing biological changes that enables it to go through the stages of invasion, extravasation to the bloodstream, seeding and colonization of host organs [113,114]. Despite these findings, more studies are still needed to understand the role of PaCSCs in the CTC population and during the formation of secondary tumors in distant organs. 

Lastly, while the tumor itself can regulate the drivers and determinants of EMT and stem-like phenotypes, the TME also constitutes an important critical factor, providing cues to the CSC subpopulation to upregulate EMT and stemness. Sainz et al. [115,116] discovered a positive feedback loop by which tumor-associated macrophages (TAMs) secrete free ISG15, which in turn mediates the reinforcement of the stemness, EMT phenotype and migratory capacity of PaCSCs. These changes were reflected in elevated mRNA levels of the pluripotency-associated genes *KLF4*, *SOX2* and *NANOG*, modulation of EMT-related genes including E-cadherin (*CDH1*), *ZEB1* and *VIM*, increased mobility assessed in migration assays, and activation of pERK1/2, involved in cell survival. In turn, CSCs were found to secrete IFN-β, inducing further expression of ISG15 by TAMs, as well as TGF-β1, Nodal and Activin that polarize macrophages towards a pro-tumor M2-like phenotype (Figure 3). As mentioned above, ISG15 is an ubiquitin-like modifier that covalently links to cytosolic and nuclear proteins by a process known as ISGylation. Higher levels of ISGylated proteins were found after treating PaCSC with recombinant ISG15, as well as enhanced phosphorylation of ERK1/2. Moreover, our group has recently discovered that CSC ISGylation levels correlate with their stem-like features, including their tumorigenic capacity, and that knockdown of ISG15 alters pancreatic CSC functions, such as EMT (unpublished data). Indeed, high ISG15 levels correlate with poor overall survival in PDAC, and ISG15 levels have been related to tumorigenesis, tumor progression and metastasis in other malignancies such as prostate or breast cancer [117,118].

### 2.3. Plasticity

During the development of adult life, there are cellular subpopulations that have the capacity to differentiate/transition into multiple lineages, which is termed “phenotypic plasticity”. While this feature is essential during embryogenesis, it is limited in adult stem cells but can be reactivated in specific tissues such as the liver, pancreas and colon as a way of maintaining or repairing the organ. Likewise, in cancer, tumor cells reactivate plasticity as a means to boost tumor progression [33]. Our understanding of normal cellular plasticity has grown over the past two decades. We now appreciate that daughter cells, progenitor cells, transient cells and even differentiated cells can re-enter the linear and hierarchical process of cellular differentiation when necessary. This ability to re-enter what was believed to be a static uni-directional process of differentiation is known as cellular plasticity. For example, upon liver damage, terminally differentiated hepatocytes can re-enter the cell cycle to replace lost hepatocytes or liver tissue without becoming full-fledged stem cells [119]. Acinar cells of the pancreas also have the ability to transdifferentiate. Reddy et al. [120] in 1984 and Dabeva et al. [121] in 1995 showed that foci of hepatic cells can appear in the pancreas of rats treated with ciprofibrate, a peroxisome proliferator, or exposed to a low-copper diet, respectively. We now recognize that acinar cells are extremely plastic and can undergo epithelial-to-epithelial transition or EMT and transdifferentiate into ductal, endocrine and even adipocytes depending on specific signals and/or microenvironmental cues [122,123]. Thus, as a whole, plasticity may be more common and much more important than previously appreciated. 

**Figure 4:**
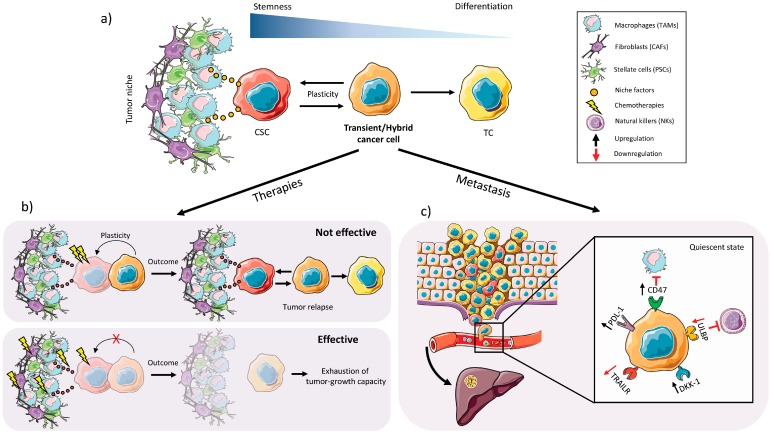
Redefining the roles of pancreatic cancer cells. (**a**) Inside the PDAC tumor we can find different types of tumor cells (TC), which have different states of differentiation and stemness: CSCs receive stimuli from the tumor niche that can influence the biology and overall status of the tumor as a whole and help CSCs maintain their stemness. In turn, CSCs can enter into an intermediate state of differentiation in which they maintain some of their stem capacity and express typical markers of differentiated TCs (hybrid/transient cancer cells). (**b**) Transient/hybrid cancer cells are able to survive under certain conditions and to regenerate tumor heterogeneity under therapeutic insult. For this reason, if combination therapies with anti-CSC/anti-tumor niche/anti-transient cell efficacy are used, it would be possible to eradicate the tumor in a more effective way. (**c**) Moreover, transient cancer cells are able to survive in the bloodstream and metastasize (e.g., to the liver) due to upregulation of CSC metastasis receptors such as CXCR4 and immune evasion receptors (PDL-1, CD47, DKK-1) and downregulation of death receptors (TRAILR and ULBP) and enter into a quiescent and immune evasion state.

In the context of cancer, it is believed that the CSC is the main source of phenotypic plasticity within a tumor, and CSC phenotypic plasticity allows for the regeneration of each cellular phenotype and subtype found within the tumor [124]. For example, within identical genetic clones we can find different CSCs that differ at the phenotypic and epigenetic levels and can give rise to multiple different subpopulations within a single tumor [125,126]. Non-CSCs, such as transient cells, likely also have a certain degree of cellular plasticity and can also contribute to tumor cell and CSC “regeneration” through a differentiation process allowing them to regain a certain level of “stem cell status” (Figure 4a). These partially differentiated transient subpopulations that retain some stem cell characteristics are more likely to act as the cells that regenerate more undifferentiated stem cell subpopulations, rather than terminally differentiated cancer cells; however, whether terminally differentiated cancer cells have no cellular plasticity is still under debate. Nevertheless, the concept that the CSC subpopulation can be replenished by non-CSCs was experimentally addressed in two recent studies targeting the Lgr5^+^ CSC population in colon cancer organoid cultures. Specifically, Shimokawa et al. [127] eliminated the Lgr5^+^ CSC population in organoids of human colorectal cancer by inserting an inducible version of the suicide-gene caspase 9 (iCasp9) into the *LGR5* locus. Upon dimerizer-mediated Lgr5 ablation, the growth of xenografts derived from the implanted organoids was reduced. In a genetically engineered mouse model approach, Melo et al. [128] used mouse Apc^min^; Kras^G12V^; p53^CRIPSR^; SMAD4^CRIPSR^ organoid cultures engineered to express the diphtheria-toxin (DT) receptor in Lgr5^+^ cells. Upon treatment with DT, organoid-derived tumor growth was halted in vivo. In both studies, however, upon removal of the dimerizer or DT, tumors regrew and the Lgr5^+^ population re-emerged, indicative of cellular plasticity driven by a Lgr5^−^ non-CSC (or quiescent CSC) transient population.

Apart from transdifferentiation, we also appreciate and understand that cancer cell plasticity encompasses many other biological processes. For example, the capacity of a cell to alter its metabolism, resist chemotherapeutic insults and even evade the immune system by upregulating immune evasion receptors or checkpoint inhibitors can each be considered a type of cellular plasticity (Figure 4b,c). One of the most well-studied types of phenotypic plasticity is EMT, described above and in references [129,130]. As mentioned earlier, recent studies have broadened the concept of phenotypic plasticity by suggesting that in cancer there is a direct link between plasticity, stemness, and EMT [106,131], demonstrating that cancer cells exist across a spectrum of E to M states, such as a hybrid EMT phenotype/state [106] with high phenotypic plasticity and stem cell-like properties. Thus, the idea that EMT transcription factors function predominantly to promote invasion, migration and dissemination is perhaps an archaic concept that should be reevaluated, particularly in the context of cancer. A plethora of recent studies demonstrate that EMT transcription factors affect many biological processes, including therapeutic resistance and metabolism, and it has also been shown that the EMT hybrid phenotype can act as an indicator of poor prognosis in different tumor types, including lung cancer [132]. As mentioned above, in 2015 two independent studies challenged the idea that EMT is necessary for metastasis. Zheng et al. [92] showed that genetic elimination of the EMT transcription factors Twist1 and Snail1 in mouse models of PDAC had no effect on metastasis to secondary organs, but were critical for chemoresistance. Likewise, Fischer et al. [133] showed in a multi-color MMTV-PyMT-based model of multifocal breast adenocarcinoma that EMT is not required for lung metastasis but contributes to chemoresistance. Thus, EMT was determined to be dispensable for metastasis but necessary for chemoresistance. A study by Biddle et al. [134] demonstrated a similar chemoresistance link by showing that both therapeutic resistance and tumor-initiating capacity in oral squamous cell carcinoma are maintained by CSCs with an EMT hybrid state. These EMT hybrid plastic CSCs were defined as CD44^high^ EpCAM^low/−^ CD24^+^. More recently, however, Brabletz and colleagues have “rectified” the issue of EMT in PDAC metastasis by showing that EMT transcription factors are not all the same and “there is variability and specificity (and not redundancy) in the role and function of different EMT transcription factors”. Not only did they re-establish the important role for EMT in PDAC metastasis, but more importantly they showed that regulatory differences exist among different EMT transcription factors and some EMT factors, such as Zeb1, play a major role in metastasis as well as cellular plasticity, specifically metabolic plasticity [93]. The sum of these new findings offers new links between EMT, stemness and plasticity at the level of hierarchy, bidirectional differentiation, chemoresistance and metabolism. Plasticity also plays a very important role in the process of MET. Once at the metastatic site, plasticity allows for restoration of the cellular heterogeneity and epithelial characteristics of the primary tumor [135]. Thus, regarding EMT and plasticity, the regeneration of CSCs from non-CSCs may occur more frequently than previously thought, and EMT transcription factors likely play a very important role in this interconversion. Of course, this transition severely hinders the study and isolation of these highly plastic CSCs as their presence within a tissue and phenotypic profile are surely susceptible to environmental and TME stimuli and may differ at any given time during the evolution of the tumor; however, new methodologies that use specific signaling mechanisms to enrich for and maintain these plastic cells in culture [134] may prove very useful for dissecting the underlying drivers of their plasticity and for therapeutic screening in drug discovery platforms.

One way in which CSCs maintain their plasticity is through their chromatin structure. Evidence suggests that CSCs may use bivalent chromatin that is found in a relatively open structure but is not transcribed until correct transcription factors are present [136]. Bivalent chromatin is different from standard chromatin (heterochromatin), which is essentially irreversibly locked and usually imposed during development [137]. It has been shown that genes strongly associated with EMT often exist in a bivalent chromatin state in cancer cells, such as prostate cancer and breast cancer cells, allowing for greater plasticity of gene expression [136,138]. For example, in breast cancer, Chaffer et al. [136] showed that the *ZEB1* promoter is maintained in a bivalent chromatin configuration in non-CSCs, which enables these cells to rapidly respond to EMT-inducing signals, such as TGF-β, from the microenvironment. There are also necessary epigenetic and metabolic modifications that facilitate phenotypic plasticity, and these modifications can be activated during different cancer processes. It is believed that some tumor cells, mainly CSCs, undergo epigenetic reprogramming to induce metabolic and phenotypic changes that are often associated with tumor invasiveness and chemoresistance [139]. Such plasticity would afford these cells a greater adaptability to challenges presented during drug therapy and present within the TME (e.g., hypoxia and oxidative stress) [126,140]. As mentioned above, we recently showed that PaCSCs upregulate the expression of the DNA methyltransferase DNMT1 promoting the hypermethylation of the CSC genome, and pharmacologic or genetic targeting of DNMT1 reduced overall methylation and severely affected CSC plasticity [78].

In addition, some CSC subpopulations with phenotypic plasticity may be found in a quiescent stage, that is, in a relatively inactive state [141] until they are required to rebuild into the primary tumor, or establish the secondary tumor after the metastatic process (Figure 4c). During this period, these cells can evade anti-tumor therapies or even the immune system. The ability to adopt a resting or quiescent state is a hallmark of plastic CSCs and is often responsible for disease relapse years after successful surgical intervention or tumor free survival. Thus, the identification of DC-TICs by Dieter et al. [28], described above, may have actually been the identification of plastic resting CSCs. Likewise, Rhim et al. [142] showed in a mouse model of PDAC that tagged PDAC cells invaded and entered the bloodstream before tumorigenesis, and these cells exhibited EMT and stem cell properties, suggesting that quiescent plastic CSCs are already present but resting/latent during early stages of disease development. Of course, for these cells to survive they must also evade the immune system. To link immune cell evasion and CSC plasticity, Malladi et al. [143] showed that “latency competent” CSCs with an inherent ability to form secondary tumors reduce the regulation of natural killer cell activator molecules when they transition into a quiescent state, through a mechanism involving autocrine inhibition of Wnt/β-catenin signaling by Dickkopf-related protein 1 (DKK1), thus avoiding death by cytolysis [143]. Thus, not only do CSCs use cellular plasticity to regulate their cell proliferation to survive in a latent state for long periods of time or to evade chemotherapeutics that target proliferating cells, but they can also regulate the expression of immune evasion receptors to avoid immune cell killing and elimination. It is still to be determined the extent to which PaCSCs also possess immune cell evasion plasticity. It was recently shown by Cioffi et al. [144] that PaCSCs upregulate the macrophage “don’t eat me signal” CD47, which affords PaCSCs protection from macrophage-mediated phagocytosis. Whether PaCSCs can also regulate the expression of T-cell checkpoint inhibitors or NK cell ligands or interacting receptors still remains to be fully elucidated. Nonetheless, not only have plastic CSCs become a very attractive target for cancer therapy, but also their presence and state within the tumor may one day be informative when evaluating patient prognosis [134].

### 2.4. CSC Autophagy and Glucose Metabolism

PDAC is characterized by a nutrient-poor and highly hypoxic microenvironment [145]. The lack of oxygen and carbon sources, such as glucose and glutamine, create an inhospitable environment where the supplies available are incapable of satisfying the requirements of the tumor cells. As such, PDAC cells and PaCSCs need to adapt to their environment by activating pathways that make them resistant to these environmental challenges [146]. For example, reduction of oxygen causes stabilization of the transcription factor HIF-1 [147], which leads to tumor cells overcoming oxygen and nutrient deprivation [148,149]. In fact, HIF-1 has been shown to suppress pyruvate dehydrogenase activity, redirecting glucose into glycolysis rather than the TCA cycle to more rapidly meet the energy requirements of the cell [150]. At the same time, hypoxia has been shown to induce a stem phenotype in many different cancers via diverse mechanisms [151,152,153], including activation of autophagy [154]. Autophagy is an adaptive catabolic process commonly activated in cells that enter into a resting and/or non-dividing state as a consequence of different environmental stressors such as lack of nutrients, depletion of growth factors and hypoxia [155]. In cancer, deregulated autophagy has been shown to be necessary and its inhibition can affect tumor growth and progression in different tumor types, including PDAC [156]. Autophagy mediates the recycling of the cell’s own components and, therefore, provides the cell with nutrients [157] due to the formation of autophagosomes. These autophagosomes are vesicles with a double membrane that engulf intra-cellular cargo such as protein aggregates, organelles and ribosomes, and they eventually fuse with the lysosome to promote the degradation of the cargo and reuse of the broken down products [155]. Autophagy signaling is mediated by the expression of Beclin1, Atg3 and conversion of the LC3B-I protein to LC3B-II [158]. LC3 processing results in its insertion into the extending phagophore membrane facilitating the engulfment of selective targets for degradation, followed by autophagosome: lysosome fusion and proteolytic degradation of the engulfed cargo.

Importantly, in PDAC, high levels of autophagy correlate with poor prognosis [159], and autophagy has been shown to be important for many aspects of CSCs biology, including survival, quiescence and EMT [160]. Compared to non-CSCs, CSCs contain more advanced autophagic vesicles and express more genes related to autophagy [154]. Thus, CSCs have been reported to be in a continuous and active autophagic state (Figure 3), and inhibition of autophagy can severely impair CSCs. For example, Yang et al. [59] showed a high correlation between autophagy and PaCSC biomarkers such as ALDH1, CD44 and CD133 in tissues. When autophagy was blocked, they saw an important reduction in the PaCSC population suggesting that autophagy is essential for the maintenance of PaCSCs. In addition, Zhu et al. [161] described that increased autophagic flux is associated with a high expression of HIF-1, and the interaction of both factors results in a dynamic equilibrium between CSCs and non-CSCs. 

Moreover, basal levels of autophagy have been shown to be necessary to maintain the pluripotency of CSCs and this process is regulated by Nicotinamide phosphoribosyltransferase (NAMPT; limiting enzyme in the NAD^+^ synthesis pathway) and the transcription factor POU5F1/OCT4 [162]. Elimination of NAMPT and POU5F1 causes a decrease in the expression of pluripotency-associated genes and upregulates markers of differentiation. Interestingly, the regulatory effects of NAMPT and POU5F1 on pluripotency are accompanied by different levels of autophagy. For example, elimination of NAMPT promotes autophagy and the elimination of POU5F1 inhibits autophagy [162]. Most importantly, any deviation from the autophagic “baseline”, either increasing or decreasing, strongly impairs CSC pluripotency and promotes differentiation and/or senescence of CSCs [163]. Taken together, these results reveal a link between the NAD^+^ biosynthesis pathway, the POU5F1 transcription factor and pluripotency, as well as identify autophagy as a new regulator of CSC pluripotency [162].

Apart from autophagy, under adverse and hypoxic microenvironmental conditions, metabolic reprogramming can also occur. Specifically, cells can increase glucose metabolism through glycolysis (Warburg effect) to meet their energy requirements; however, glycolysis may not fully cover the metabolic needs of pancreatic tumors due to their low energetic efficiency [164,165]. In fact, mutations in the *KRAS* gene have been shown to lead to a distinct metabolic program in pancreatic cancer cells characterized by increased glycolytic flow, providing tumor cells with proliferative substrates and a sufficient energy supply [166]. In situations where this increase in metabolic rate cannot be effective, the cell requires adequate mitochondrial function for the energy supply required for its survival [166].

Despite recent efforts to unravel the metabolic characteristics of pancreatic tumors, little is known about the metabolic phenotype of PaCSCs. Recently, Sancho et al. [167] observed that unlike their differentiated cellular progeny, PaCSCs are highly dependent on mitochondrial oxidative phosphorylation (OXPHOS) (Figure 3). The OXPHOS system constitutes the main source of energy in differentiated cells in adult tissues [168], and interestingly this pathway seems to be the preferred mechanism for energy production in CSCs in various types of tumors [169], including PDAC [167]. This dependence is mediated by the Peroxisome proliferator-activated receptor γ co-activator 1 α (PGC-1α) transcription factor, which plays an important role in cellular metabolic energy regulation. In turn, PGC-1α is regulated by the expression of the c-MYC oncogene, which is expressed at low to undetectable levels in PaCSCs [167]. 

Although the OXPHOS system involves a significantly greater number of biochemical reactions, it is almost 20 times more efficient in terms of generation of ATP per unit of glucose. It is hypothesized that the dependence of PaCSCs on OXPHOS may reflect an adaptation of these cells to the nutrient-poor microenvironment of pancreatic tumors [154]. Because CSCs are virtually dependent on mitochondrial function, mitochondria may be an excellent target for therapeutic purposes. In fact, several investigations have shown that there is a greater sensitivity to chemotherapies if they include mitochondrial inhibitors such as metformin [170]. As mentioned above, Krebs et al. [93] also showed that Zeb1 is involved in the metabolic plasticity of PDAC cells derived from KPC mice. While the levels of Zeb1 specifically in PaCSCs were not analyzed, this multifunctional EMT transcription factor may also be involved in PaCSCs OXPHOS.

## 3. Conclusions

PDAC presents a very important global therapeutic healthcare challenge. At present, little progress has been made to advance the diagnosis and treatment of this disease. Current research in the field of cell biology, genetics, metabolism and immunology have created new hope for developing new tools for early diagnosis and for improving treatment of this disease; however, more research to better understand the biology of PDAC is still needed. Along these lines, our ever-expanding understanding of the PaCSC population and the role these cells play in all aspects of PDAC is promising. We now know that PaCSCs plays a fundamental role in the initiation and development of PDAC, and these cells are largely responsible for the aggressive, chemoresistant and metastatic nature of this cancer. While this review did not delve in great depth into the importance of the TME, it is critical that we do not ignore the other non-cancer cells (e.g., CAFs, PSCs and TAMs) that communicate with the PaCSC. Likewise, the extracellular matrix proteins, the architecture and stiffness of the matrix itself are also likely to play a determining role in PaCSC processes and the PaCSC state. Thus, understanding the communication network(s) that exists within the TME, including the CSC niche, are not only important, but may also be relevant at the level of resistance to conventional therapies and cellular plasticity. Regarding the latter, the latest insights into tumor cell plasticity have unveiled that CSCs are not an entity but rather a state that can fluctuate throughout the evolution of the tumor. In fact, recent studies published this year indicate that non-CSCs, such as transient amplifying, hybrid or progenitor daughter cells, can replenish the CSC pool even after their efficient elimination, highlighting a previously unappreciated and enormous therapeutic challenge. Thus, the more we know about what makes a CSC a CSC, how the TME influences the state of these cells, and what are the key drivers of PaCSC plasticity, the closer we will be to developing therapies that disrupt the state of the CSC and simultaneously target those cells that can replenish the CSC compartment.
